# Macroalgal browsing on a heavily degraded, urbanized equatorial reef system

**DOI:** 10.1038/s41598-017-08873-3

**Published:** 2017-08-21

**Authors:** Andrew G. Bauman, Andrew S. Hoey, Glenn Dunshea, David A. Feary, Jeffrey Low, Peter A. Todd

**Affiliations:** 1 Experimental Marine and Ecology Laboratory, Department of Biological Sciences, National University of Singapore, Singapore, 117543 Singapore; 2 ARC Centre of Excellence for Coral Reef Studies, James Cook University, Townsville, Queensland 4811 Australia; 3 Ecological Marine Services, 2/3 Thomsen St, Millbank, QLD 4670 Australia; 4 Natural History Museum of Denmark, University of Copenhagen, Øster Voldgade 5-7, 1350 Copenhagen, Denmark; 5 Fish Ecology Research Group, School of Life Sciences, University of Nottingham, Nottingham, NG7 2RD United Kingdom; 6 National Biodiversity Centre, National Parks Board, 1 Cluny Road, Singapore, 259569 Singapore

## Abstract

The removal of macroalgal biomass is critical to the health of coral reef ecosystems. Previous studies on relatively intact reefs with diverse and abundant fish communities have quantified rapid removal of macroalgae by herbivorous fishes, yet how these findings relate to degraded reef systems where fish diversity and abundance are markedly lower and algal biomass substantially higher, is unclear. We surveyed roving herbivorous fish communities and quantified their capacity to remove the dominant macroalga *Sargassum ilicifolium* on seven reefs in Singapore; a heavily degraded urbanized reef system. The diversity and abundance of herbivorous fishes was extremely low, with eight species and a mean abundance ~1.1 individuals 60 m^−2^ recorded across reefs. Consumption of *S*. *ilicifolium* varied with distance from Singapore’s main port with consumption being 3- to 17-fold higher on reefs furthest from the port (Pulau Satumu: 4.18 g h^−1^; Kusu Island: 2.38 g h^−1^) than reefs closer to the port (0.35–0.78 g h^−1^). Video observations revealed a single species, *Siganus virgatus*, was almost solely responsible for removing *S*. *ilicifolium* biomass, accounting for 83% of the mass-standardized bites. Despite low herbivore diversity and intense urbanization, macroalgal removal by fishes on some Singaporean reefs was directly comparable to rates reported for other inshore Indo-Pacific reefs.

## Introduction

Coral reefs are among the world’s most threatened coastal marine ecosystems. Local anthropogenic stressors (e.g. overfishing, coastal development and pollution), coupled with increasing effects of climate change, have caused regional declines in live coral cover (e.g., Caribbean^1^ and Great Barrier Reef ^2^). Collectively, these external impacts can alter the balance between primary producers and consumers resulting in some reefs being overgrown by fleshy macroalgae (or seaweed)^3–5^. The removal of macroalgal biomass from reefs is, therefore, considered a critical process in preventing, and potentially reversing macroalgal-dominance^4, 6, 7^, and thereby promoting coral dominance on tropical reefs^8^. Numerous studies have shown that the removal of large fleshy macroalgae that dominate on degraded reefs (e.g., *Sargassum*) is restricted to a limited suite of fish species: the macroalgal browsers^9–11^.

Research into the ecology of macroalgal browsing fishes (hereafter ‘browsers’) has provided numerous insights into the process of macroalgal removal on coral reefs (see review^12^). For example, it has become evident that browsers are highly selective, feeding on a relatively small subset of available macroalgal species^13, 14^ or even components of individual macroalga^15^, and that the process of macroalgal removal varies across a range of spatial (e.g. regional^16^; reefs^17^; habitats^18^; sites^19^), and over temporal scales (e.g. seasonal^20^). Additionally, the consumption of fleshy macroalgae at any one location is often dominated by a limited number of species at any time^10, 11, 21, 22^, despite a range of browsers being present^12^. To date, the vast majority of studies that have directly quantified macroalgal browsing on coral reefs have been conducted on reef systems in regions with relatively diverse and abundant herbivorous fish populations^12^. Consequently, it remains unclear how these findings relate to heavily degraded, urbanized reef systems where the diversity and abundance of herbivorous fishes are typically lower, and macroalgal biomass substantially higher.

Biodiversity is an important component of any ecosystem, and many studies have described positive relationships between species diversity and ecosystem functions^23, 24^. The mechanistic bases for these relationships are hypothesized to be related to functional redundancy, where multiple species perform similar ecosystem roles so that losses of single species may be compensated for by increased contributions of other species (i.e. insurance hypothesis)^25, 26^ or by dampening fluctuations of individual species abundances that perform particular ecological functions (i.e. portfolio effect^27^). These relationships, however, are contingent upon the diversity of responses among different species to environmental change or disturbance (response diversity)^28^. Collectively, biological diversity is hypothesized to ensure continuity of ecosystem processes following environmental change^29^ by increasing the probability of including species that contribute disproportionately to certain functions, and/or increasing the probability of multiple species contributing to the same processes^30^. However, our understanding of the potential relationship between biodiversity and macroalgal removal on coral reefs is limited because most macroalgal removal studies have been conducted on reefs with relatively high species richness. Given that the number of people living adjacent to reefs is increasing^31^, it is essential to understand if and how browser diversity influences rates of macroalgal removal in increasingly urbanized environments.

Coral reefs surrounding Singapore provide an ideal system to examine how reefs function within a heavily urbanized environment. Extensive coastal and port development, coupled with shipping activities have resulted in over 80% of Singapore’s coastline being modified^32^, as well as high levels of sedimentation, turbidity and eutrophication that well exceed thresholds of most tropical reefs^33^. Collectively, these impacts have reduced total coral reef area in Singapore from 17 km^2^ to 9.5 km^2, 32^. Singapore’s reefs have also been impacted by climate change, with severe thermal bleaching events in 1998 and 2010^34^, and a mild bleaching event in 2013^35^. Despite these adverse conditions, coral communities are relatively diverse (250 species^36^) and abundant (~36% cover across shallow reef crest^37^) compared to other Indo-Pacific reefs (e.g. Great Barrier Reef mean coral cover ~23%^2^). In contrast, Singaporean reef fish communities are relatively depauperate with less than 200 total species and few recorded herbivorous species^38^. The objectives of this study were to: (1) quantify spatial variation in the removal of *Sargassum* among reefs with differing coral and macroalgal cover spanning the southern islands of Singapore, and (2) determine whether the fish species responsible for removing macroalgal biomass changes significantly over these scales.

## Results

### Benthic communities

**Figure 1:**
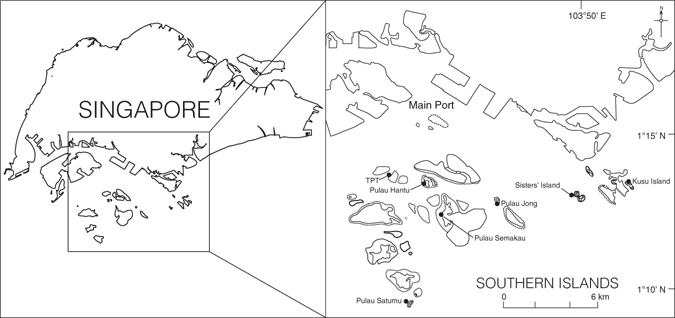
Map showing seven reefs in the southern islands of Singapore. Reefs organized from west to east: Terumbu Pempang Tengah (TPT), Pulau Satumu, Pulau Hantu, Pulau Semakau, Pulau Jong, Sisters’ Island and Kusu. Dotted lines represent fringing reef areas. The map is modified from Bauman *et al*.^37^ and used with permission of the author (https://creativecommons.org/licenses/by/4.0/).

**Figure 2:**
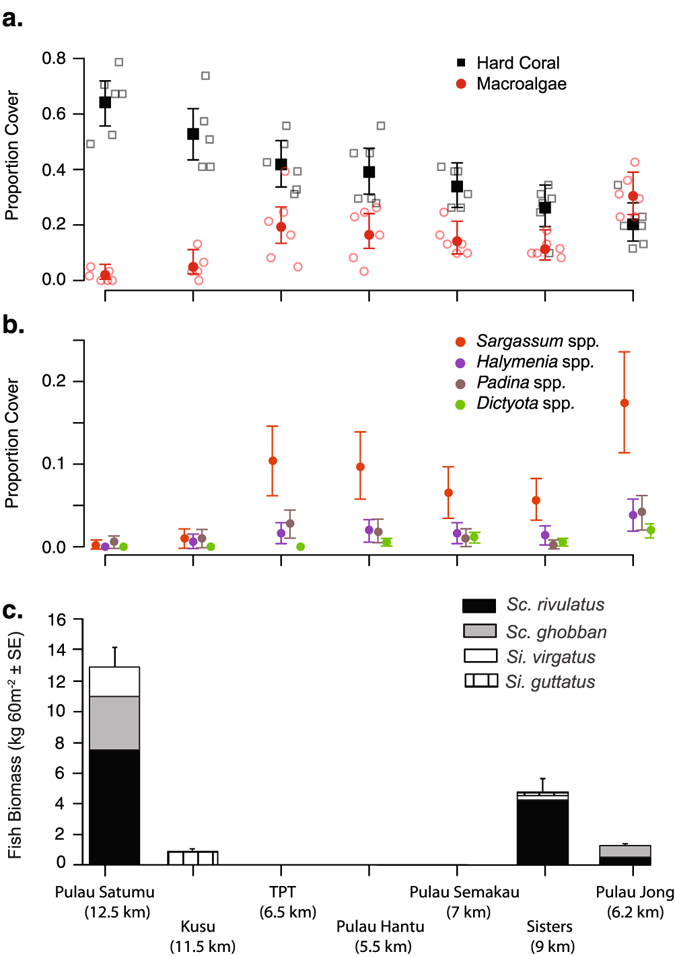
Variation in the proportion of hard coral and macroalgal benthic cover at each reef, ranked by proportion of hard coral cover along the x-axis (decreasing left to right), and browsing fish biomass among reefs in Singapore. (**a**) Raw data (open symbols) and model fits (filled symbols ± 95% CI) from separate binomial GLM’s for hard coral and macroalgae cover. (**b**) Model fit ( ±95% CI) from multivariate model of macroalgae genera point-intercept counts per transect, presented as proportion cover. Estimates from macoalgae genera with significant differences between reefs are presented. (**c**) Macroalgal browser biomass estimates for each reef based on six 30 × 2-m belt transects. Distance (km) from the main port is presented in parentheses.

Live coral and macroalgae dominated benthic communities of the seven reefs surveyed in Singapore (Supplementary Table S1), but varied among reefs (Figs 1 and 2a, Supplementary Table S2). Coral cover was highest on reefs furthest from the main port of Singapore, Pulau Satumu and Kusu (Fig. 2a), and lowest on Pulau Jong and Sisters’ Island (Fig. 2a). Conversely, macroalgal cover was highest on reefs closer to the port (Pulau Jong, TPT and Pulau Hantu, Fig. 1) and lowest on Pulau Satumu and Kusu (Fig. 2a). Macroalgal community composition differed significantly among reefs (macroalgal composition _MVAbund_ *p* = 0.0001, Fig. 2b, Supplementary Table S3). *Sargassum* spp. was the most abundant macroalgae across reefs (51.5% ± 1.1% SE) with the highest densities found on Pulau Jong and lowest on Pulau Satumu and Kusu Island (Fig. 2b).

### Roving herbivore abundance and biomass

Eight roving herbivorous fish species, from three families (Labridae, Siganidae, and Pomacanthidae), were recorded across both the belt transects and timed swims (see Supplementary Table S4). The mean abundance and biomass of roving herbivorous fishes across reefs in the belt transects was low with 1.1 ± 0.4 individuals 60 m^−2^ and 0.48 kg 60 m^−2^, respectively (Fig. 2c). The highest fish richness and total roving herbivore biomass was recorded on Pulau Satumu furthest from the port (~12.5 km), while no roving herbivores were recorded on the three reefs closest to the port (i.e., Pulau Hantu, Pulau Semakau and TPT, Fig. 2c). *Scarus rivulatus* were the most abundant roving herbivore accounting for 51% and 62% of the total abundance and biomass across reefs, respectively (Fig. 2c, Supplementary Table S4). *Scarus ghobban* and *Siganus virgatus* were the next most abundant herbivores, each with seven individuals recorded for each species, and accounted for 21.1% and 10.8% of the total herbivore biomass (Supplementary Table S4).

### Macroalgal removal

**Figure 3:**
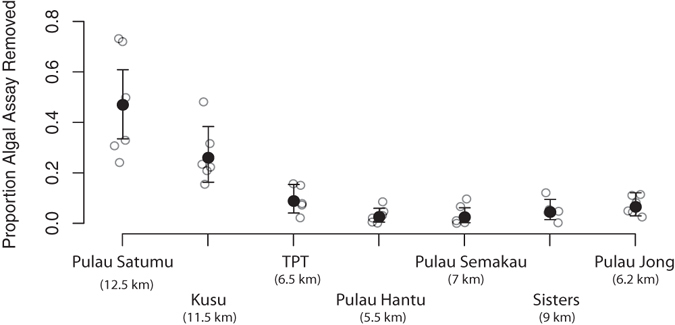
Proportion of algal assay mass removed on each reef (*n* = 5–6). Raw data (open symbols) and back-transformed model fits (filled symbols ± 95% CI) from linear model of logit-transformed proportion mass removed from each assay. Reefs are ranked by proportion of hard coral cover along the x-axis (decreasing left to right) and distance from the main port presented in parentheses (km).

There were marked differences in the removal rates of *S*. *ilicifolium* among Singapore’s reefs (*F*_(6,32)_ = 16.37, *p* < 0.001) ranging from 2.9% 4.5 h^−1^ (1.26 g, 4.5 h^−1^) on Pulau Hantu to 47.3% 4.5 h^−1^ (18.6 g, 4.5 h^−1^) on Pulau Satumu (Fig. 3, Supplementary Fig. S1). Rates of macroalgal removal were positively related to distance from Singapore’s main port. Removal rates of *S*. *ilicifolium* on reefs furthest from the port (Pulau Satumu and Kusu) were 5–17 and 3–9 fold higher than all other reefs (Fig. 3).

### Video analysis

**Figure 4:**
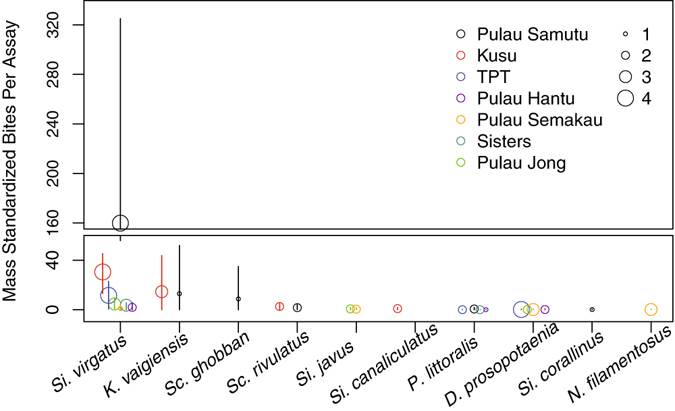
Mean number of mass standardized bites recorded per assay (*n* = 4) for each fish species at each reef. Symbol size indicates the number of video assays the species was observed feeding on at each reef, and whiskers represent the data range. Species are ranked by total mass standardized bites along the x-axis (decreasing left to right).

A total of 5,302 bites (1,023 mass-standardized bites, henceforth ‘ms bites’) from 10 fish species were recorded on the *S*. *ilicifolium* assays across reefs (Fig. 4). Feeding rates on the *S*. *ilicifolium* differed significantly among reefs (_MVAbund_ *p* = 0.01, Fig. 4), with feeding rates generally increasing with distance from Singapore’s main port. There was substantially higher feeding on the two reefs furthest from the port, Pulau Satumu (733 ms bites) and Kusu (196 ms bites), compared to all other reefs combined (total 95 ms bites). A single species, *S. virgatus*, was responsible for the majority of the feeding, accounting for 83.0% of the ms bites (849 ms bites), and was recorded feeding on every reef and on 21 of the 28 filmed assays (Fig. 4). *Kyphosus vaigiensis* was the only other browser recorded to take a substantial number of bites from *S*. *ilicifolium*, accounting for 10.7% of the total ms bites (110 ms bites). However, *K*. *vaigiensis* was only recording feeding on four assays across two reefs (Pulau Satumu and Kusu). The remaining five herbivorous fish species identified in videos, including the browsers *Siganus* *canaliculatus* and *Siganus* *javus*, accounted for 5.8% of ms bites (Fig. 4).

## Discussion

Macroalgal removal across reefs in Singapore showed a high degree of spatial variation consistent with patterns reported from other Indo-Pacific reef systems^10, 18, 19, 21, 39, 40^. Overall, the loss of macroalgal biomass within assays due to browsers in Singapore was low (0.35–4.18 g h^−1^) compared to offshore reef studies on the GBR (2–100 g h^−1^)^41^ and Ningaloo (42–53 g h^−1^)^42^, but was directly comparable to rates reported from coastal reefs in the Seychelles (0.28–10 g h^−1^)^40^ and nearshore reefs on the GBR (0.34–4.29 g h^−1^)^10, 19, 39^ using similar methods. Interestingly, the current study revealed that a single species was almost solely responsible for macroalgal biomass removal in Singapore. The barhead spinefoot, *S*. *virgatus*, was the most dominant consumer of *S*. *ilicifolium* assays accounting for the majority (83%) of mass standardized bites. Despite the limited diversity and abundance of herbivorous fishes, poor water quality and large-scale urbanization on Singaporean reefs, a key ecosystem process (i.e., browsing of canopy-forming *Sargassum*) appears to be maintained on some reefs.

Spatial variation in removal rates among reefs appeared to be related to the distance from Singapore’s main port, with removal rates being 3- to 17-fold higher on the two reefs furthest from the port (Pulau Satumu and Kusu) than reefs closer to the port. Previous macroalgal removal studies report similar spatial patterns of increasing, but variable, removal rates with increasing distance from shore. For example, a study on the GBR revealed distinct cross-shelf gradient with highest removal rates of *Sargassum* on the mid-shelf reefs^41^. Similarly, rates of macroalgal removal on Indonesian reefs have been reported to be positively related to distance from shore^21^. The consistency of these inshore-offshore (or cross-shelf) gradients suggests removal rates may be related to changes in water quality^21^, and/or benthic and fish community structure with distance from shore^43^. For example, coral cover generally increases^43–45^ and macroalgal cover decreases with increasing distance from shore^41, 46^. Similarly within Singapore, reefs closer to the port have less abundant and diverse coral^36, 44^ and fish^47^ communities, and higher macroalgal cover compared to reefs further from the port^48^.

High macroalgal cover is known to negatively affect removal rates^41, 49^ with evidence suggesting browsers have a reduced ability to control macroalgae once it becomes abundant^49^. Browsers may avoid dense areas of macroalgae^49^, or assays may be less apparent on reefs with high macroalgae abundance^41, 46^. The highest removal rates in this study were on reefs with the lowest macroalgal cover, and vice versa, suggesting that local macroalgal communities may be inducing similar negative responses in local browser assemblages. In contrast, on the reefs with low macroalgal cover (i.e. Pulau Satumu and Kusu) transplanted assays are likely to be more apparent^41^ and may attract browsers from beyond their normal foraging range^40^, thereby increasing removal rates. Alternatively, spatial variability in removal rates may be simply due to spatial differences in local browser abundances among reefs that were not fully captured during the underwater visual census (UVC). Previous studies have shown that many browsers are averse to divers^10, 41^, and as such their densities are typically under-estimated during visual diver surveys. This may be further compounded by low visibility on most of Singapore reefs (<3 m)^50^.

Results from the video analysis revealed a limited suite of browsers feeding on *Sargassum* within Singapore waters. Of the 10 fish species recorded feeding on macroalgal assays, four are recognized browsers and have been recorded to feed on *Sargassum* elsewhere (*S. virgatus*, *S*. *canaliculatus*, *S*. *javus* and *K. vaigiensis*)^12^. *S*. *virgatus*, appeared to be the most functionally important browser across all reefs, irrespective of macroalgal cover or distance from Singapore’s port. *S*. *virgatus* has been identified as a key browser on coastal reefs in Sulawesi^21^, and its sister species, *Siganus doliatus*, has frequently been reported as a key browser removing *Sargassum* and other macroalgal species from inshore reefs of the GBR^15, 19, 51^ and on fringing reefs in Ningaloo^42^. Whether these feeding patterns for *S*. *virgatus* are maintained over longer temporal scales remains to be seen and will require further investigation. In addition to *S*. *virgatus*, there were two other browsing rabbitfishes, namely *S*. *canaliculatus* and *S*. *javus* observed feeding on *S*. *ilicifolium* in Singapore, although their contribution was minimal (~1% of all bites). Collectively, these observations provide further evidence of the importance of rabbitfishes in removing macroalgae, and add to a growing body of literature describing the importance of rabbitfishes to ecological processes in shallow marine habitats both within their historic range^6, 10, 19, 22, 52^ and recently expanded ranges^53, 54^.

Despite reefs around Singapore supporting a low abundance and diversity of herbivorous fishes, the consumption of *Sargassum* biomass in this study was directly comparable to inshore reefs in other regions using similar methods, where species richness, abundance and biomass of herbivorous fishes are considerably higher^10, 19, 21, 39, 40^. Further, the apparent reliance on a single browser (*S*. *virgatus*) for the removal of *Sargassum* on Singaporean reefs is similar to suggestions from species rich coral reef ecosystems such as the GBR^9, 11^. If our results, and those of previous studies using similar methods, are representative of the rates and agents of macroalgal removal over longer temporal scales, then collectively these studies suggest that browsing may be largely independent of local biodiversity. Biodiversity has long been argued to ensure continuity of ecosystem functions by increasing the number of species contributing to any particular function (i.e., functional redundancy^24, 25^) and increasing the diversity of response of species within a functional group to a given disturbance (i.e., response diversity^26, 28^). Although further research is required to investigate the relationship between biodiversity and browsing on coral reefs, results from short-term studies of macroalgal removal suggest there is limited redundancy in macroalgal browsing both within and among spatial scales, and therefore browsing may be sensitive to the loss of single species. Previous studies have reported similar patterns for external bioerosion on coral reefs, with rates of bioersion being sensitive to the loss of a single species, *Bolbometopon muricatum* ^55, 56^.

While the results of the present study suggest the rates of macroalgal removal on Singaporean reefs are comparable to those from other, less degraded Indo-Pacific coastal reef systems^10, 18, 19, 39–42^, caution should be used if extrapolating our findings across broader spatial and temporal scales. The present study was conducted over a period of three weeks, with individual assays exposed to local herbivore assemblages for 4.5 hrs. Although most recent studies of macroalgal removal on coral reefs have used similar methods and sampling designs^10, 19, 39, 40, 42, 46^, most fail to capture any variation across longer temporal scales. Of the few studies that have investigated temporal variation in macroalgal removal, Mantyka and Bellwood^51^ reported no differences between morning and afternoon deployments, while Lefevre and Bellwood^20^ reported seasonal differences in both the rates and agents (i.e., species responsible) of macroalgal removal on an inshore reef of the GBR. Further, our results only relate to the removal of adult macroalgal biomass and do not consider other ecological processes that may influence macroalgal biomass over the entire life cycle of *Sargassum* ^20^ or density-dependent processes that may affect removal.

In summary, the observed patterns in macroalgal removal have important implications for Singapore’s coral reefs. Despite a low diversity and abundance of herbivores, and browsers in particular, and the impacted state of the reefs surrounding Singapore, a key ecological process (i.e. macroalgal browsing) is maintained at rates comparable to less impacted coastal reef systems. The apparent reliance on a single species (*S*. *virgatus*), however, suggests that the removal of macroalgal biomass in Singapore may be sensitive to fluctuations in the population size of this species. Moreover, low removal rates and high macroalgal cover recorded on reefs closest to Singapore’s port, likely as a result of reduced nearshore water quality, could make some reefs in Singapore more vulnerable to future disturbance events. Further studies are required to elucidate the mechanisms that regulate the process of macroalgal removal across degraded reef systems such as Singapore, and whether these processes change over environmental gradients and temporal scales.

## Methods

### Study location

This study was conducted in August 2015 among the southern islands of Singapore (1°17′N, 103°36′E; Fig. 1). Seven reefs were selected across a range of distances (~5 –13 km) from the main port of Singapore to examine spatial variation in the rates of macroalgal removal and to identify the fish species responsible for consuming the macroalgal biomass. Adjacent reefs were separated by ~ 2–5 km. Within each reef, benthic and fish assemblages, and rates of macroalgal removal were quantified on the reef crest (3–4 m). The reef crest was selected as it generally supports the greatest abundance and diversity of herbivorous fish and has the highest rates of herbivory on Indo-Pacific reefs^17, 18^. Further, most coral cover in Singapore is limited to a relatively narrow strip between the reef crest and upper reef slope from 3–6 m depth^36, 57^. This depth restriction is primarily due to the upper reef flats (0–2 m) being dominated by fleshy macroalgae for most of the year (e.g. *Sargassum* ^48^), and extreme light attenuation with increasing depth (>6 m) from chronic high sediment deposition and suspended particles^35^.

### Benthic surveys

Benthic communities at each reef were quantified using six non-overlapping 30-m point-intercept transects. Transects were laid parallel to the reef crest (3–4 depth) and live benthos and abiotic substratum immediately under the transect tape was recorded at 0.5 m intervals along each transect, giving a total 61 points per transect (366 points per site). Live benthos were identified as macroalgae (>10 mm in height), live scleractinian (hard) coral, epilithic algal matrix (EAM), crustose coralline algae (CCA), alcyonacean (soft) coral, and other living benthic organisms (“others”). Abiotic substratum categories included unconsolidated rubble, sand and dead coral. All macroalgae and live scleractinian corals were identified to the genus level.

### Distribution of roving herbivorous fishes

To characterize fish communities within each reef, and to quantify the distribution and abundance of all roving herbivorous and nominally herbivorous fishes (i.e. Acanthuridae, Kyphosidae, Labridae (parrotfishes), Pomacanthidae, and Siganidae) a combination of belt transects and timed swims were conducted. Within each reef all fishes >5 cm were visually censured within six 30 × 2-m belt transects along the reef crest (2–3 m depth). All fishes were identified to species and categorized in 5 cm interval size classes (total length). Density estimates were later converted to biomass using published species length-weight relationships^52, 58^.

To augment fish community surveys, and due to generally poor diving conditions (visibility <3 m), 20-min timed-swims were used to survey more mobile roving herbivorous fish species. A diver (always ASH) swam at a constant speed along a depth contour and recorded all nominally herbivorous fishes greater than 10 cm total length (TL) within 2.5 m either side the path swam^11^. Due to the small size of the reefs it was only possible to conduct a single timed swim within each of two habitats the reef crest (2–3 m) and the reef flat (1–2 m).

### Macroalgal removal and herbivore feeding activity

A series of standardized macroalgal assays were used to quantify variation in the removal of macroalgae by herbivorous fishes among reefs. *Sargassum ilicifolium* was selected for the assays, as it is the most abundant macroalgal species found on reefs in Singapore^48^. *Sargassum ilicifolium* of similar sizes were collected daily from the reef flat at Pulau Hantu (Fig. 1). Individual *S*. *ilicifolium* thalli were spun in a salad spinner for 20 s to remove excess water, weighed (mean 40.3 ± 6.2 g) and placed into individually labeled plastic bags. Four *S*. *ilicifolium* were subsequently transplanted to the reef crest at each reef site, and replicated over two non-consecutive days. Each *S*. *ilicifolium* assay was attached directly to the reef with a rubber band and short length of galvanized wire (0.5 mm diameter) wrapped around the holdfast, and secured to the reef with a galvanized nail. To control for any losses due to handling one assay was placed within a cylindrical exclusion cage (10 cm radius, 100 cm height, 0.5 cm mesh). The reduction in total biomass of *S*. *ilicifolium* assays among reefs within the exclusion cages was low (overall mean = 0.8 g 4.5 h^−1^). All assays were individually identified with a plastic label attached to the reef and adjacent assays at each site were separated by a minimum of 5 m. All assays were deployed between 0900 and 1100 hrs and collected after approximately 4.5 h. After collection, each assay was spun and re-weighed as described previously.

To identify the herbivorous fish species responsible for removing *S*. *ilicifolium* biomass, small stationary underwater video cameras (GoPro) were used to record the daily feeding activity on two uncaged assays deployed within each site. Each camera, attached to a lead weight (2 kg), was positioned approximately 1 m away from each assay. Filming commenced immediately after each assay was attached to the reef, with a small-scale bar positioned adjacent to the assay for approximately ~10 s to enable calibration of fish sizes on the video footage. Filming was continuous for the entire 4.5 h deployment period, resulting in 18 h (2 × 4.5 hrs of footage d^−1^) of video observations for each reef (126 h in total).

All video footage was viewed and the number of bites taken from the *S*. *ilicifolium* by each species and size (total length, TL) was recorded. Size estimates for each species were converted to biomass using published length-weight relationships^52, 58^. To account for variation in the impact of individual bites due to body size and the amount of algal mass removed per bite a mass standardized bite impact was calculated as the product of body mass (kg) and number of bites (following Hoey and Bellwood^11^).

### Data analysis

All data analyses were performed in R^59^. Variation in the proportion of *S*. *ilicifolium* assays removed among reefs was assessed using linear models. After controlling for handling loss by subtracting the mean value of loss from control (caged) assays from each treatment (following methods described by Cronin and Hay^60^), the proportions of biomass from each assay were logit transformed^61^ and the effects of assay day and site were assessed by backward model selection of linear models using F-tests of nested models. There was no evidence of any effect of day, therefore the final model included only reef. Post-hoc multiple comparisons for all linear and generalized linear models were performed using the package *multcomp* ^62^ applying single step Tukey adjustments to *p*-values. In order for individual assays on each day to be considered independent, we assumed that the functional response of the browser community to individual thalli was independent of the feeding history of the individuals present.

The proportions of hard coral and macroalgal cover from benthic surveys were analysed with binomial generalized linear models (GLM). Overall significance of the terms in the final models was calculated using analysis of deviance. To analyze reef differences in macroalgal communities (from transect data) and active herbivorous fish community feeding rates (from video assays), a model based approach for analyzing multivariate abundance data was used (*mvabund* package^63^). Each *mvabund* model provides an overall *p*-value for the multivariate test (the effect of reef) and univariate tests for the difference of each group between reefs, with *p-*values calculated using PIT-trap resampling (i.e. probability integral transform residual bootstrap) to account for correlation in testing and adjusted for multiple testing using a step-down resampling procedure^64^. For analysis of herbivorous fish community feeding rates, mass standardized bites for each fish species for each assay was rounded to whole integers and modeled using a negative binomial error structure. For the macroalgae community analysis, a negative binomial count error structure was applied to transect-point counts of each macroalgae group. Although the macroalgae data was strictly proportional (i.e. it has an upper limit of the number of points in the transect), the majority of macroalgal group point intercept counts were between zero and three (~92%), and model diagnostics showed an appropriate mean-variance response for a negative binomial distribution. Assumptions of all models were validated using standard residual, normal Q-Q and mean-variance diagnostic plots.

## Electronic supplementary material

Supplementary Information

## References

[1] Gardner TA, Côté IM, Gill JA, Grant A, Watkinson AR (2003). Long-term region-wide declines in Caribbean corals. Science.

[2] Hughes TP (2017). Global warming and recurrent mass bleaching of corals. Nature.

[3] Hughes TP (1994). Catastrophes, phase shifts and large-scale degradation of a Caribbean coral reef. Science.

[4] McClanahan TR, Muthiga NA, Mangi S (2001). Coral and algal changes after the 1998 coral bleaching: Interaction with reef management and herbivores on Kenyan reefs. Coral Reefs.

[5] Cheal AJ (2010). Coral-macroalgal phase shifts or reef resilience: links with diversity and functional roles of herbivorous fishes on the Great Barrier Reef. Coral Reefs.

[6] McCook LJ (1999). Macroalgae, nutrients and phase shifts on coral reefs: scientific issues and management consequences for the Great Barrier Reef. Coral Reefs.

[7] Hughes TP (2007). Phase shifts, herbivory and the resilience of coral reefs to climate change. Curr. Biol..

[8] Bellwood DR, Hughes TP, Folke C (2004). & Nyström Confronting the coral reef crisis. Nature.

[9] Bellwood DR, Hughes TP, Hoey AS (2006). Sleeping functional group drives coral-reef recovery. Curr. Biol..

[10] Fox RJ, Bellwood DR (2008). Remote video bioassays reveal the potential feeding impact of the rabbitfish *Siganus canaliculatus* (f. Siganidae) on an inner-shelf reef on the Great Barrier Reef. Coral Reefs.

[11] Hoey AS, Bellwood DR (2009). Limited functional redundancy in a high diversity system: single species dominates key ecological process on coral reefs. Ecosystems.

[12] Puk LD, Ferse SCA, Wild C (2016). Patterns and trends in coral reef macroalgae browsing: a review of browsing herbivorous fishes of the Indo-Pacific. Rev. Fish Biol. Fisheries.

[13] Mantyka CS, Bellwood DR (2007). Macroalgal grazing selectivity among herbivorous coral reef fishes. Mar. Ecol. Prog. Ser..

[14] Rasher DB, Hoey AS, Hay ME (2013). Consumer diversity interacts with prey defenses to drive ecosystem function. Ecology.

[15] Streit RP, Hoey AS, Bellwood DR (2015). Feeding characteristics reveal functional distinctions among browsing herbivorous fishes on coral reefs. Coral Reefs.

[16] Hay ME (1984). Patterns of fish and urchin grazing on Caribbean coral reefs: are previous results typical. Ecology.

[17] Hoey AS, Bellwood DR (2010). Among habitat variation in herbivory on Sargassum spp. on a mid-shelf reef in the northern Great Barrier Reef. Mar. Biol..

[18] Fox RJ, Bellwood DR (2007). Quantifying herbivory across a coral reef depth gradient. Mar. Ecol. Prog. Ser..

[19] Loffler Z, Bellwood DR, Hoey AS (2015). Among-habitat algal selectivity by browsing herbivores on an inshore coral reef. Coral Reefs.

[20] Lefèvre CD, Bellwood DR (2011). Temporal variation in coral reef ecosystem processes: herbivory of macroalgae by fishes. Mar. Ecol. Prog. Ser..

[21] Plass-Johnson JG, Ferse SCA, Jompa J, Wild C, Teichberg M (2015). Fish herbivory as a key ecological function in a heavily degraded coral reef system. Limnol. Oceanogr..

[22] Gilby BL, Tibbets IR, Stevens T (2016). Low functional redundancy and high varability in *Sargassum* browsing fish populations in a subtropical reef system. Mar. Freshwater Res..

[23] Loreau M (2001). Biodiversity and ecosystem functioning: current knowledge and future challenges. Science.

[24] Hooper DU (2005). Effects of biodiversity on ecosystem functioning: a consensus of current knowledge. Ecol. Monogr..

[25] Walker BH (1992). Biodiversity and ecological redundancy. Conserv. Biol..

[26] Mori AS, Furukawa T, Sasaki T (2012). Response diversity determines the resilience of ecosystems to environmental change. Biological Reviews.

[27] Tilman D (1996). Biodiversity: population versus ecosystem stability. Ecology.

[28] Elmqvist T (2003). Response diversity, ecosystem change, and resilience. Front. Ecol. Environ..

[29] McCann KS (2000). The diversity-stability debate. Nature.

[30] Loreau M, Hector A (2001). Partitioning selection and complementarity in biodiversity experiments. Nature.

[31] Mora C (2011). Global human footprint on the linkage between biodiversity and ecosystem functioning in reef fishes. PLoS Biol..

[32] Lai S, Loke LHI, Hilton MJ, Bouma TJ, Todd PA (2015). The effects of urbanization on coastal habitats and the potential for ecological engineering: a Singapore case study. Ocean Coast. Manage..

[33] Browne NK, Tay JKL, Low J, Larson O, Todd PA (2015). Fluctuations in coral health of four common inshore reef corals in response to seasonal and anthropogenic changes in water quality. Mar. Environ. Res..

[34] Guest JR (2012). Contrasting patterns of coral bleaching susceptibility in 2010 suggest an adaptive response to thermal stress. PLoS ONE.

[35] Chou LM (2016). Differential response of coral assemblages to thermal stress underscores the complexity in predicting bleaching susceptibility. PLoS ONE.

[36] Huang DW, Tun KPP, Chou LM, Todd PA (2009). An Inventory of zooxanthellate scleractinian corals in Singapore including 33 new records. The Raffles Bulletin of Zoology.

[37] Bauman AG (2015). Coral Settlement on a Highly Disturbed Equatorial Reef System. PloS ONE.

[38] Lim, K. K. P. & Low, J. K. Y. A guide to common marine fishes of Singapore. *Singapore Science Centre* (1998).

[39] Cvitanovic C, Bellwood DR (2009). Local variation in herbivore feeding activity on an inshore reef of the Great Barrier Reef. Coral Reefs.

[40] Chong-Seng KM, Nash KL, Bellwood DR, Graham NAJ (2014). Macroalgal herbivory on recovering versus degrading coral reefs. Coral Reefs.

[41] Hoey AS, Bellwood DR (2010). Cross-shelf variation in browsing intensity on the Great Barrier Reef. Coral Reefs.

[42] Michael PJ, Hyndes GA, Vanderklift MA, Vergés A (2013). Identity and behaviour of herbivorous fish influence large-scale spatial patterns of macroalgal herbivory in a coral reef. Mar. Ecol. Prog. Ser..

[43] Wismer S, Hoey AS, Bellwood DR (2009). Cross-shelf benthic community structure on the Great Barrier Reef: a relationships between macroalgal cover and herbivore biomass. Mar. Ecol. Prog. Ser..

[44] Dikou A, van Woesik R (2006). Survival under chronic stress from sediment load: Spatial patterns of hard coral communities in the southern islands of Singapore. Marine Pollution Bulletin.

[45] Hennige SJ (2010). Acclimation and adaptation of scleractinian coral communities along environmental gradients within an Indonesian reef system. J. Exp. Mar. Biol. Ecol..

[46] Vergés A, Vanderklift MA, Doropoulos C, Hyndes GA (2011). Spatial patterns in herbivory are influenced by structural complexity but not by algal traits. PLoS ONE.

[47] Low, J. K. Y. & Chou, L. M. Distribution of coral reef fish in Singapore. In: *Marine Science:**Living Coastal**Resources* (eds Chou, L. M. & Wilkinson, C. R.) **6**, 139–144 (Third ASEAN Science and Technology Work Conference Proceedings, 1992).

[48] Low, J. K. Y. *Sargassum* on Singapore’s reefs. *Ph*.*D*. *Thesis*, National University of Singapore (2014).

[49] Hoey AS, Bellwood DR (2011). Suppression of herbivory by macroalgal density: a critical feedback on coral reefs?. Ecol. Lett..

[50] Chou LM (1996). Response of Singapore reefs to land reclamation. Galaxea.

[51] Mantyka CS, Bellwood DR (2007). Macroalgal grazing selectivity among herbivorous coral reef fishes. Mar. Ecol. Prog. Ser..

[52] Hoey AS, Brandl SJ, Bellwood DR (2013). Diet and cross-shelf distribution of rabbitfishes (f. Siganidae) on the northern Great Barrier Reef: implications for ecosystem function. Coral Reefs.

[53] Vergés A (2014). The tropicalization of temperate marine ecosystems: climate-mediated changes in herbivory and community phase shifts. Proc. R. Soc. Lond. [Biol].

[54] Vergés A (2016). Long-term empirical evidence of ocean warming leading to tropicalization of fish communities, increased herbivory, and loss of kelp. Proc. Natl. Acad. Sci. USA.

[55] Bellwood DR, Hoey AS, Choat JH (2003). Limited functional redundancy in high diversity systems: resilience and ecosystem function on coral reefs. Ecol. Lett..

[56] Bellwood DR, Hoey AS, Hughes TP (2012). Human activity selectively impacts the ecosystem roles of parrotfishes on coral reefs. Proc. R. Soc. Lond. [Biol].

[57] Guest JR (2016). 27 years of benthic and coral community dynamics on turbid, highly urbanised reefs off Singapore. Scientific Reports.

[58] Kulbicki M, Guillemot N, Amand M (2005). A general approach to length-weight relationships for New Caledonian lagoon fishes. Cybium.

[59] R Core Team. R: a language and environment for statistical computing. R Foundation for Statistical Computing. Vienna, Austria (2016).

[60] Cronin G, Hay ME (1996). Susceptibility to herbivores depends on recent history of both the plant and animal. Ecology.

[61] Warton D, Hui FK (2011). The arcsine is asinine: the analysis of proportions in ecology. Ecology.

[62] Hothorn T, Bretz F, Westfall P (2008). Simultaneous inference in general parametric models. Biometric Journal.

[63] Wang Y, Naumann U, Wright ST, Warton D (2012). I. mvabund- an R package for model-based analysis of multivariate abundance data. Methods Ecol. Evol..

[64] Westfall, P. H. & Young, S. S. Resampling-based multiple testing: examples and methods for p-value adjustments. *John Wiley & Sons*, New York, New York (1993).

