# Supplementary material for: Macroalgal browsing on a heavily degraded, urbanized equatorial reef system
Source: Sci Rep. 2017 Aug 21;7:8352. doi: 10.1038/s41598-017-08873-3 (PMC5567118; doi:10.1038/s41598-017-08873-3)
Supplement: Supplementary file 1 — Supplementary Information [file 41598_2017_8873_MOESM1_ESM.doc]

Macroalgal browsing on a heavily degraded, urbanized equatorial reef system

AG Bauman, AS Hoey, G Dunshea, DA Feary, Jeffrey Low, PA Todd

**Supplementary Information**

**Results**


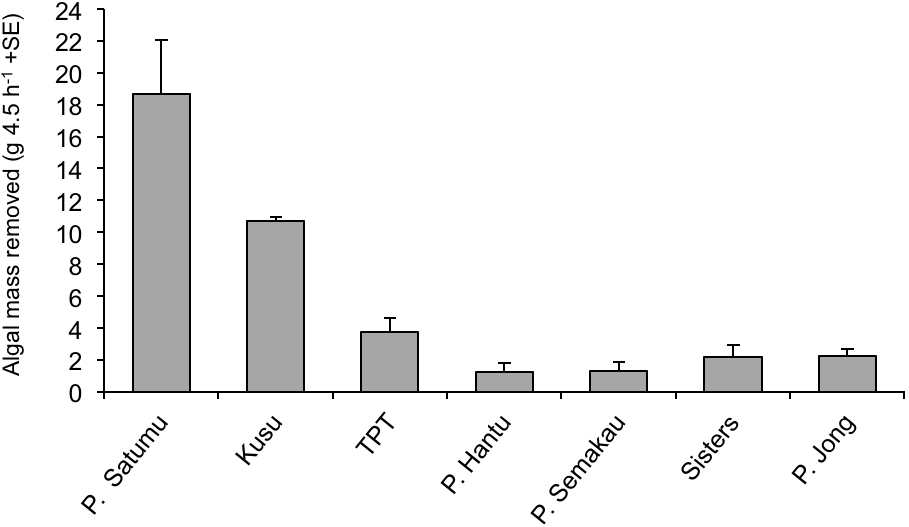


Fig. S1. Variation in the total biomass removed from *Sargassum ilicifolium* assays among reefs in the southern islands of Singapore.

Table S1. Summary of benthic community composition for the seven reefs in southern Singapore. Reefs are ranked by proportion of hard coral cover (decreasing left to right) Mean percent cover of each of the substrata categories based on six replicate 30 m transects within each reef. Values in parentheses are standard errors (± SE).

|  | Pulau Satumu | Kusu Island | TPT | Pulau Hantu | Pulau Semakau | Sisters’ Island | Pulau Jong |
| --- | --- | --- | --- | --- | --- | --- | --- |
| Live hard coral | 64.2 (4.6) | 52.8 (6.1) | 41.8 (3.9) | 39.1 (4.8) | 33.9 (2.8) | 26.2 (3.5) | 20.2 (3.6) |
| Macroalgae | 1.6 (0.8) | 5.2 (2.2) | 19.1 (5.1) | 16.9 (3.8) | 14.5 (2.3) | 11.7 (1.4) | 30.9 (3.1) |
| Chlorophyta |  |  |  |  |  |  |  |
| *Bryopsis* sp. | 0 | 1.3 (0.6) | 0.5 (0.3) | 0.5 (0.5) | 0.5 (0.4) | 2.1 (0.7) | 0.5 (0.2) |
| *Halimeda* sp. | 0 | 0 | 0.3 (0.3) | 0.3 (0.3) | 0.5 (0.4) | 0.3 (0.3) | 0.3 (0.2) |
| Rhodophyta |  |  |  |  |  |  |  |
| *Halymenia sp.* | 0 | 0.7 (0.4) | 1.6 (0.9) | 1.9 (1.1) | 1.6 (0.7) | 1.4 (0.5) | 3.8 (0.4) |
| *Hypnea* sp. | 0 | 0.6 (0.4) | 1.1 (0.6) | 1.4 (0.9) | 1.1 (0.6) | 0.6 (0.4) | 0.8 (0.3) |
| Ochrophyta |  |  |  |  |  |  |  |
| *Dictyota* sp. | 0 | 0 | 0 | 0.5 (0.3) | 1.1 (0.4) | 0.5 (0.4) | 1.9 (0.3) |
| *Padina* sp. | 0.5 (0.4) | 1.0 (0.6) | 2.7 (1.0) | 1.9 (0.8) | 1.1 (0.6) | 0.3 (0.3) | 4.1 (0.4) |
| *Lobophora* sp. | 0.8 (0.6) | 0.3 (0.3) | 0.8 (0.4) | 0 | 1.6 (0.8) | 0.3 (0.3) | 1.9 (0.3) |
| *Sargassum* sp. | 0.3 (0.3) | 1.0 (0.6) | 10.4 (2.9) | 9.8 (2.5) | 6.6 (1.8) | 5.7 (0.9) | 17.5 (1.4) |
| *Turbinara* sp. | 0 | 0 | 0.3 (0.3) | 0.3 (0.3) | 0 | 0 | 0 |
| Unknown macroalgae | 0 | 0.3 (0.3) | 1.4 (0.7) | 0.3 (0.3) | 0.3 (0.3) | 0.5 (0.4) | 0 |
| Epilithic algal matrix | 12.6 (2.7) | 7.5 (1.5) | 18.0 (1.3) | 11.7 (2.2) | 13.1 (2.7) | 11.2 (1.9) | 12.6 (1.3) |
| Crustose coralline algae | 0 | 0 | 0 | 0 | 0 | 0 | 0 |
| Sponge | 0.5 (0.3) | 1.6 (0.5) | 2.5 (0.6) | 3.0 (0.7) | 1.9 (1.3) | 4.1 (1.5) | 1.4 (0.5) |
| Soft coral | 0 | 2.3 (0.9) | 0.3 (0.3) | 0 | 0.5 (0.5) | 0.3 (0.3) | 0.3 (0.3) |
| Rubble | 10.6 (1.7) | 14.4 (1.1) | 9.0 (1.8) | 10.9 (2.6) | 11.7 (1.6) | 23.0 (3.2) | 18.0 (2.4) |
| Sand | 5.5 (1.6) | 7.9 (3.3) | 5.5 (1.6) | 16.7 (3.4) | 19.7 (2.9) | 15.6 (1.0) | 12.6 (2.0) |
| Dead coral & rock | 3.3 (0.7) | 1.0 (0.6) | 1.9 (1.3) | 0.3 (0.3) | 1.6 (0.8) | 6.0 (1.3) | 2.2 (0.8) |
| Cyanobacteria | 0 | 1.3 (1.0) | 0 | 0 | 0.8 (0.5) | 1.4 (0.8) | 0.3 (0.3) |
| Other live | 1.4 (0.8) | 5.9 (4.3) | 1.9 (0.9) | 1.4 (1.1) | 2.2 (0.4) | 0.5 (0.4) | 1.4 (0.7) |

Table S2. Summary of final model results of hard coral and macroalgal cover, evaluated by binomial generalized linear models (GLMs) and significance calculated using analysis of deviance.

| **Model** | **Δdeviance** | **Δdf** | **Scaled deviance** | ***p*** |
| --- | --- | --- | --- | --- |
| **Hard coral cover** | | | | |
| Full model | 96.812 |  |  |  |
| Reef | 306.263 | 6 | 75.241 | 3.42E-14*** |
|  | | | | |
| **Macroalgal cover** | | | | |
| Full model | 95.291 |  |  |  |
| Reef | 270.007 | 6 | 66.388 | 2.25E-12*** |

Table S3: Summary statistics for analysis of macroalgal communities across sites. Multivariate negative binomial count model applied to macroalgae count data from six replicate 30 m transects and univariate tests for the difference of each group between sites. Bold color indicates significance (*p* < 0.05).

Multivariate test:

|  | **Res.Df** | **Df.diff** | **Dev** | **Pr(>Dev)** |
| --- | --- | --- | --- | --- |
| (Intercept) | 40 |  |  |  |
| reefs | 34 | 6 | 169.7 | **0.001** |

Univariate test:

|  | **Dev** | **Pr(>Dev)** |
| --- | --- | --- |
| (Intercept) |  |  |
| *Bryopsis*-*Chlorodesmis* spp. | 13.703 | 0.175 |
| *Caulerpa* spp. | 0 | 1 |
| *Dictyota* spp. | **20.291** | **0.025** |
| *Halimeda* spp. | 4.333 | 0.84 |
| *Halymenia* spp. | **20.335** | **0.025** |
| *Hypnea* spp. | 7.371 | 0.66 |
| *Lobophora* spp. | 15.066 | 0.137 |
| *Padina* spp. | **20.975** | **0.023** |
| *Sargassum* spp. | **51.607** | **0.001** |
| *Turbinaria* spp. | 4.915 | 0.84 |
| Unknown | 11.098 | 0.32 |

Table S4. Summary of individual fish counts and biomass for belt transects, and individual fish counts for timed swims methods. Bold color indicates total abundance and biomass for all reefs and individual reefs.

| Site | P. Satumu | Kusu Is. | TPT | P. Hantu | Semakau | Sister’s Is | P. Jong | Total |
| --- | --- | --- | --- | --- | --- | --- | --- | --- |
| **Belt transects** | | | | | | | | |
| Counts |  |  |  |  |  |  |  |  |
| *Siganus virgatus* | 5 | 0 | 0 | 0 | 0 | 2 | 0 | **7** |
| *Siganus guttatus* | 0 | 3 | 0 | 0 | 0 | 2 | 0 | **10** |
| *Scarus ghobban* | 2 | 0 | 0 | 0 | 0 | 0 | 3 | **5** |
| *Scarus rivulatus* | 11 | 0 | 0 | 0 | 0 | 11 | 5 | **23** |
| **Total roving herbivores** | **18** | **3** | **0** | **0** | **0** | **15** | **8** | **44** |
|  |  |  |  |  |  |  |  |  |
| Biomass (kg) |  |  |  |  |  |  |  |  |
| *Siganus virgatus* | 1.92 | 0 | 0 | 0 | 0 | 0.27 | 0 | **2.19** |
| *Siganus guttatus* | 0.26 | 0.98 | 0 | 0 | 0 | 0.27 | 0 | **1.51** |
| *Scarus ghobban* | 3.51 | 0 | 0 | 0 | 0 | 0 | 0.75 | **4.26** |
| *Scarus rivulatus* | 7.48 | 0 | 0 | 0 | 0 | 4.25 | 0.50 | **12.23** |
| **Total biomass** | **13.17** | **0.98** | **0** | **0** | **0** | **4.79** | **1.25** | **20.19** |
|  |  |  |  |  |  |  |  |  |
| **Timed Swim Surveys** | | | | | | | | |
| *Siganus virgatus* | 9 | 3 | 0 | 0 | 0 | 3 | 1 | **16** |
| *Siganus guttatus* | 0 | 0 | 0 | 0 | 0 | 0 | 0 | **0** |
| *Siganus javus* | 0 | 2 | 0 | 0 | 0 | 0 | 2 | **4** |
| *Siganus punctatus* | 0 | 1 | 0 | 0 | 0 | 0 | 0 | **1** |
| *Scarus ghobban* | 4 | 10 | 0 | 0 | 0 | 1 | 1 | **16** |
| *Scarus rivulatus* | 34 | 48 | 0 | 0 | 0 | 22 | 10 | **114** |
| *Scarus niger* | 2 | 0 | 0 | 0 | 0 | 0 | 0 | **2** |
| *Pomacanthus sexstriatus* | 2 | 0 | 2 | 0 | 0 | 0 | 0 | **2** |
| **Total roving herbivores** | **51** | **64** | **2** | **0** | **0** | **26** | **14** | **157** |
